# The biological classification of mental disorders (BeCOME) study: a protocol for an observational deep-phenotyping study for the identification of biological subtypes

**DOI:** 10.1186/s12888-020-02541-z

**Published:** 2020-05-11

**Authors:** Tanja M. Brückl, Victor I. Spoormaker, Philipp G. Sämann, Anna-Katharine Brem, Lara Henco, Darina Czamara, Immanuel Elbau, Norma C. Grandi, Lee Jollans, Anne Kühnel, Laura Leuchs, Dorothee Pöhlchen, Maximilian Schneider, Alina Tontsch, Martin E. Keck, Leonhard Schilbach, Michael Czisch, Susanne Lucae, Angelika Erhardt, Elisabeth B. Binder

**Affiliations:** 1 Department of Translational Research in Psychiatry, Max Planck Institute of Psychiatry, Kraepelinstr. 2-10, 80804 Munich, Germany; 2 Max Planck Institute of Psychiatry, Munich, Germany; 3 Berenson-Allen Center for Noninvasive Brain Stimulation and Division for Cognitive Neurology, Department of Neurology, Beth Israel Deaconess Medical Center, Harvard Medical School, Boston, MA USA; 4 Independent Max Planck Research Group for Social Neuroscience, Max Planck Institute of Psychiatry, Munich, Germany; 5 International Max Planck Research School – Translational Psychiatry (IMPRS-TP), Max Planck Institute of Psychiatry, Munich, Germany; 6 Department of Psychiatry and Behavioral Sciences, Emory University School of Medicine, Atlanta, USA

**Keywords:** Translational, Transdiagnostic, Psychiatry, Research domain criteria (RDoC), Neuroimaging, Omics, Biology-based taxonomy, Stress, Depression, Anxiety

## Abstract

**Background:**

A major research finding in the field of Biological Psychiatry is that symptom-based categories of mental disorders map poorly onto dysfunctions in brain circuits or neurobiological pathways. Many of the identified (neuro) biological dysfunctions are “transdiagnostic”, meaning that they do not reflect diagnostic boundaries but are shared by different ICD/DSM diagnoses. The compromised biological validity of the current classification system for mental disorders impedes rather than supports the development of treatments that not only target symptoms but also the underlying pathophysiological mechanisms. The Biological Classification of Mental Disorders (BeCOME) study aims to identify biology-based classes of mental disorders that improve the translation of novel biomedical findings into tailored clinical applications.

**Methods:**

BeCOME intends to include at least 1000 individuals with a broad spectrum of affective, anxiety and stress-related mental disorders as well as 500 individuals unaffected by mental disorders. After a screening visit, all participants undergo in-depth phenotyping procedures and omics assessments on two consecutive days. Several validated paradigms (e.g., fear conditioning, reward anticipation, imaging stress test, social reward learning task) are applied to stimulate a response in a basic system of human functioning (e.g., acute threat response, reward processing, stress response or social reward learning) that plays a key role in the development of affective, anxiety and stress-related mental disorders. The response to this stimulation is then read out across multiple levels. Assessments comprise genetic, molecular, cellular, physiological, neuroimaging, neurocognitive, psychophysiological and psychometric measurements. The multilevel information collected in BeCOME will be used to identify data-driven biologically-informed categories of mental disorders using cluster analytical techniques.

**Discussion:**

The novelty of BeCOME lies in the dynamic in-depth phenotyping and omics characterization of individuals with mental disorders from the depression and anxiety spectrum of varying severity. We believe that such biology-based subclasses of mental disorders will serve as better treatment targets than purely symptom-based disease entities, and help in tailoring the right treatment to the individual patient suffering from a mental disorder. BeCOME has the potential to contribute to a novel taxonomy of mental disorders that integrates the underlying pathomechanisms into diagnoses.

**Trial registration:**

Retrospectively registered on June 12, 2019 on ClinicalTrials.gov (TRN: NCT03984084).

## Background

The lack of biological validity of the current classification systems of mental disorders, namely the World Health Organization’s (WHO) International Classification of Diseases (ICD-10) [1] and the American Psychiatric Association’s Diagnostic and Statistical Manual of Mental Disorders (DSM-5) [2], is considered to be one of the major reasons why psychiatry has made little progress in translating biomedical research findings into clinical practice. The past three decades have been marked by tremendous technological advances in basic scientific disciplines such as genomics and imaging. However, unlike in other medical disciplines (e.g., oncology), these new developments have not yet reached patients with mental health problems. The rapid gain in biological knowledge has not improved the understanding, diagnosis or treatment of mental disorders (e.g. [3]).

Why is it so difficult for psychiatry to translate new (neuro)-biological research findings into clinical applications? It has been argued that our current diagnostic classification approach and the way it defines mental disorders hinders the translation of biological knowledge into the clinic [4]. Currently, the diagnosis of a mental disorder is based on predominantly self-reported symptoms (e.g., feeling sad). It does not rely on any biological or etiological information. Diagnostic criteria only require the presence of a certain number of symptoms over a defined period of time and that the symptoms cause clinically significant impairment in daily life functioning.

There are several attributes of these symptom-based disease categories that hinder causal research for mental disorders: 1) Disorder categories are not clearly separated from each other. Comorbidity is the rule and not the exception. In the Netherlands Study of Depression and Anxiety (NESDA), for example, 75% of individuals with a lifetime diagnosis of depression also met criteria for an anxiety disorder and among those with an anxiety disorder, 81% fulfilled criteria for depression [5] raising the question of whether these two disorders share common causes. 2) Different symptom profiles can lead to the same diagnosis. A patient complaining about markedly diminished interest, weight gain, hypersomnia, fatigue and diminished ability to concentrate would receive a diagnosis of major depression, as would a patient presenting an almost opposite pattern of symptoms (depressed mood, weight loss, insomnia, psychomotor agitation, feelings of inappropriate guilt, suicide attempt). 3) Assigning a mental diagnosis is a categorical yes/no decision that depends on partly arbitrary thresholds. A person reporting only four impairing depressive symptoms (instead of the required five) would not be diagnosed with depression but nevertheless share many similarities with a person meeting the threshold diagnosis. 4) Symptoms overlap between different classes of disorders. For example, psychotic features can be part of schizophrenia, major depression or bipolar disorder but the underlying pathophysiological mechanism may be the same. In sum, the high degree of comorbidity, heterogeneity, categorical threshold definitions and overlapping symptoms across disorders underline that symptom-based disorder categories do not constitute useful concepts for biomedical research into the causes of mental disorders (for an in-depth review of diagnostic systems see [6]).

Therefore, the National Institute of Mental Health (NIMH) as well as the biomedical workgroup of the EC-funded Roadmap for mental health (ROAMER) have called for new paradigms and ways to classify mental disorders in biomedical research in order to promote the goal of precision or stratified medicine in psychiatry [4, 7]. Transdiagnostic approaches in psychiatry such as the NIMH Research Domain Criteria (RDoC) have been developed in response to this call [8]. Instead of grouping patients into classes by their reported symptoms, RDoC classifies according to dysregulations in major domains of human functioning that are highly relevant for psychopathology (e.g. motivational, emotional, cognitive systems as well as social behavior) (RDoC webpage: [9]).

The term RDoC is a direct reference to RDC, the Research Diagnostic Criteria that revolutionized psychiatric classification systems in the late seventies. When the RDC were proposed [10], the most pressing problem of psychiatry was that mental diagnoses were not clearly defined. Through specifying observable symptoms and criteria for mental disorders, RDC, as all editions of the DSM thereafter [2, 11–14], helped to create a common language in psychiatry. In fact, these descriptive classification systems laid the foundation for the scientific investigation of mental disorders. Before the DSM-III [11], the first DSM edition incorporating RDC, mental disorders were ill-defined and hardly investigable phenomena. Nonetheless, the expectation that a purely descriptive classification approach that does not rely on a specific theory would lead to the identification of the neurobiological underpinnings of mental disorders has not been fulfilled, as almost four decades of research have shown. Numerous biological correlates of mental disorders are now known, but symptom-based categories of mental disorders map poorly onto dysfunctions in brain circuits or neurobiological pathways. Patients with different disease mechanisms requiring different treatments may be diagnosed with the same disorder and patients with different diagnoses may share the same pathophysiology.

While the RDC aimed at overcoming the lack of reliability of mental diagnoses, the aim of RDoC-like approaches such as the Biological Classification of Mental Disorders (BeCOME) study is to identify biology-based disease categories. The long-term aim of BeCOME is to contribute to a novel taxonomy of mental disorders that integrates the underlying pathomechanisms into diagnoses. In BeCOME, patients with various affective, anxiety and stress-related mental disorders and controls are comprehensively (neuro) biologically characterized on two consecutive days. Assessments comprise genetic, molecular, physiological, neuroimaging, neurocognitive, psychophysiological and psychometric measurements. This multilevel information set will be used to identify data-driven biologically-informed categories of mental disorders [15]. The focus of BeCOME lies on stress-related mental disorders.

## Methods

### Overview and setting

BeCOME is an observational and exploratory study that was initiated in 2015 by the Max Planck Institute of Psychiatry (MPIP) in Munich, Germany. It collates the Institute’s translational and clinical research infrastructure for the combined collection of deep phenotype and omics data in order to gain a better understanding of the biological basis of mental disorders. The core of the study consists of the comprehensive cross-sectional characterization of patients with depressive, anxiety and stress-related mental disorders and healthy individuals in basic motivational, emotional, cognitive and regulatory processes as well as stress arousal on two consecutive days. Patients who continue treatment at the MPIP (making up approximately one third of the total patient sample), are asked to take part in brief follow-up examinations around study days 14, 28 and 56, when they come in for their regular appointments, in order to evaluate the stability of basic phenotypic and biological parameters. External patients and healthy participants are not prospectively examined. For them the study ends at study day 2. The study was approved by the local Ethics Committee of the Ludwig Maximilians University, Munich, Germany, and written informed consent is obtained from all participants. Data have so far been collected only at the MPIP, but further sites may be invited to participate. The study is conducted in accordance with the Declaration of Helsinki.

### Recruitment

In order to include individuals with varying degrees and a broad range of mental disorders from the anxiety and depression spectrum and to achieve adequate participant enrolment to reach target sample size, we employ the following recruitment strategies:

1. Eligible patients seeking treatment in one of our outpatient clinics are asked to participate and informed about the study by the treating physician.
2. Additional patients are recruited through a cooperation network with surrounding psychiatric and psychotherapy practices. Our collaboration partners either distribute study flyers in their waiting rooms and/or actively address the study to eligible patients.
3. Patients and healthy volunteers are also recruited though the MPIP website providing information about BeCOME and other ongoing studies that need participants.
4. We also approach potential patients and healthy volunteers through advertisements in print/social media and distribute flyers at local mental health events, meetings of anxiety and depression self-help groups, pharmacies etc.

The way of recruitment is documented which allows for the determination of the sampling probability for each group of participants as well as the examination of systematic biases between differently recruited groups of participants. In the case of systematic biases, we will adapt our recruiting strategy accordingly and for example additionally approach individuals randomly selected from the residents’ registration office. The current sample size comprises a total of 307 participants who went through study days 1 and 2. The proportion of patients recruited through our outpatient clinic amounts to 22.5% (*N* = 91). Independent of the participants’ self-referral as patient or healthy volunteer, case status is ascertained with a fully standardized diagnostic interview (DIA-X/M-CIDI). Approximately 16% of the sample do not meet criteria for DIA-X/M-CIDI diagnosis at a subthreshold or threshold level and can be considered as super healthy controls [16].

### Screening

First contact with the study team is made either through self-referral in response to advertisements (e.g. webpage, flyers etc.) or through referral of a clinician from the MPIP clinic or a collaborating mental health practice. All patients and healthy volunteers expressing interest in participating receive (usually via email) the informed consent form which contains a detailed description of the study assessments and are asked to complete an online screening questionnaire checking eligibility criteria via secure email transfer. Since the major study assessments are rather sensitive to the influence of psychotropic substances (e.g. psychophysiological, neuroimaging and omics markers) and the study was designed to focus on neurobiological dysregulations associated with the disease status, inclusion criteria had to be kept strict in regard to the consumption of psychotropic substances (see Table 1). Any individuals with acute schizophrenia or psychotic symptoms as well as current eating disorders are excluded from the study, in order to minimize the confounding influence of medical conditions other than affective and anxiety disorders. If all criteria for study entry are met (see Table 1) and the consent form has been read, participants are invited to further appointments at the MPIP and receive their study time schedule via email.

**Table 1:** Eligibility criteria for participation in BeCOME

Inclusion criteria	Exclusion Criteria
- Aged between 18 and 75 years - No intake of any psychotropic medication/substance for a minimum of 2 months before study day 1. - For the inclusion of patients, a wide spectrum of affective, anxiety and stress-related mental disorders according to the criteria of DSM-IV or DSM-5 [2, 14] are allowed, specifically these are: Depressive disorders; Anxiety and obsessive-compulsive disorders: agoraphobia with and without panic disorder, panic disorder, social phobia, specific phobia, generalized anxiety disorder, obsessive compulsive disorder; Stress and trauma-associated mental disorders (e.g. posttraumatic stress disorder).	- Current illness in the field of organic mental disorders; - Affective disorders caused by a medical condition - Organic mental disorders (e.g. dementia) - Current disorders of schizophrenia; - Current eating disorder; - Mental retardation and profound developmental disorders; - Severe neurological or internal medical illness; - Posttraumatic or post-ischemic brain damage or elapsed cerebral hemorrhage; - Acute suicidality; - Pregnancy and postpartum period; - Magnetic resonance imaging contraindications (e.g. non-MR compatible metal implants including cardiac pacemakers, claustrophobia); - Myopia <− 6 D, which cannot be compensated by contact lenses or MR compatible glasses (Cambridge Research Systems, Rochester, UK); - Current substance abuse; - Current or past substance dependence; Risky alcohol consumption, screened with the Alcohol Use Disorder Identification Test – Consumption questions (AUDIT-C) [17] and defined as score of ≥5 in males and of ≥4 in females [18].

### Study time schedule

**Fig. 1:**
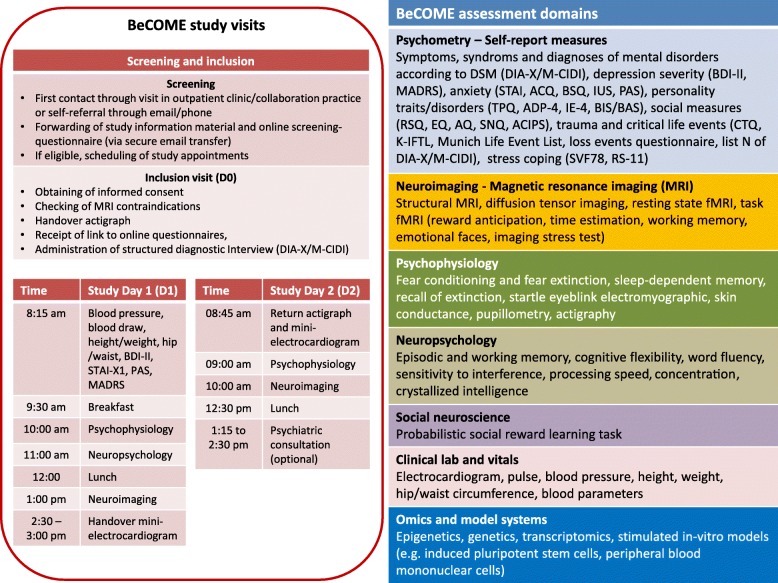
BeCOME schedule and assessment domains. Legend: DIA-X/M-CIDI, lifetime and 12-month version of the computer-assisted Munich-Composite International Diagnostic Interview; BDI-II, Beck Depression Inventory Second Edition; MADRS, Montgomery-Asberg Depression Rating Scale; STAI, State-Trait-Anxiety Inventory; ACQ, Agoraphobic Cognitions Questionnaire; BSQ, Body Sensations Questionnaire; IUS, Intolerance-of-Uncertainty Scale: PAS, Panic and Agoraphobia Scale; TPQ, Tridimensional Personality Questionnaire; ADP-IV, Assessment of DSM-IV Personality Disorders; IE-4; Internal-External Locus of Control Scale; BIS/BAS, Behavioral Inhibition and Behavioral Approach System Scales; RSQ, Relationship Scales Questionnaire; EQ, Empathy Quotient; AQ, Autism Quotient; SNQ, Social Network Questionnaire; ACIPS, Anticipatory and Consummatory Interpersonal Pleasure Scale; CTQ, Childhood Trauma Questionnaire; KIFTL, short inventory for the assessment of early traumatic life events; SVF78, German stress coping questionnaire – 78 items version; RS-11, resilience scale 11 items version

Figure 1 (left side) depicts the time schedule for a BeCOME participant. Study participation consists of three visits at the MPIP. The inclusion visit takes part approximately one to 2 weeks before study day 1. The assessment times on study day 1 and 2 are the same for all participants, while the schedule of the inclusion visit can be flexibly arranged depending on the participant’s availabilities. Approximately 2 days before each study visit, participants receive an email reminder or a phone call.

#### Inclusion visit (D0)

During the inclusion visit (D0), participants are informed in detail about the study by a staff psychologist or physician. They receive information on the purpose of the research, the study assessments, handling of data and their right to withdraw from study participation at any time. A staff physician examines whether there are any magnet resonance imaging (MRI) contraindications. When all study-related questions are clarified and the willingness for participation still exists, participants are asked for their informed written consent. For study inclusion, they need to agree to being informed in the case of medically relevant incidental blood or MRI findings that are indicative of a brain disorder, general medical problems, or an increased neurovascular risk. Study participation is not associated with any risks and not expected to produce any harms. Some of the study procedures (e.g. questions related to trauma exposure or stress test on day 2) may lead to emotional distress. Participants are informed about this and informed that they can consult a doctor or psychologist from the outpatient clinic during their study visits in case they need it (in addition to the psychiatric consultation on study day 2). Research assistants with direct patient contact are trained in how to respond to an emotional crisis and equipped with an emergency plan. In case of an emotional crisis, the participant is brought to the outpatient clinic. After participants have given their written informed consent, they are asked to wear an actigraph until the morning of study day 2. They also receive two paper and pencil questionnaires (for the assessment of sociodemographic factors and loss events) and an online link to a battery of computerized questionnaires with the request to complete these questionnaires by study day 1. The duration of the informed consent procedure is about 1 h. After a break, the fully structured diagnostic interview (DIAX/M-CIDI) is administered by a trained study assistant (approximately duration 2 to 3 h). Depending on the time schedule of the participant, the interview can also be moved to the end of study day 2.

#### Study days 1 and 2 (D1 and D2)

The major study assessments and experimental procedures take place on two consecutive days called study day 1 (D1) and study day 2 (D2). D1 takes from 08:15 am until 03:00 pm and consists of the following parts (see also Fig. 1):

- Blood draw (fasting) for clinical laboratory parameters as well as plasma and blood containing the anticoagulant ethylenediamine tetraacetic acid (EDTA) for the extraction of DNA, RNA and for the isolation of peripheral blood mononuclear cells (PBMCs). PBMCs are stored so that they can be used for the generation of induced pluripotent stem cells;
- Measurement of weight, height, waist-hip-ratio, blood pressure;
- Psychophysiology (part 1);
- Neuropsychology;
- Neuroimaging (part 1);
- For the detection of heartrate variability, a mini-electrocardiogram is applied for 18 h.

On D2 participants undergo part 2 of psychophysiology and neuroimaging. D2 ends with an optional psychiatric consultation in the MPIP outpatient clinic. All participants receive a compensation of 150 Euros for their time and effort and get vouchers for breakfast and lunches on D1 and D2.

#### Follow-up visits

Only patients who are treated at the MPIP come in for repeated blood draws (for DNA, RNA, plasma) and psychometric assessments on study days 14, 28 and 56. At 4 and 12 months after study inclusion, MPIP patients are asked for a follow up of psychometric assessments.

### Assessment domains

The study combines psychometric data with biological measures. Particular emphasis is placed on the investigation of pathophysiological changes connected to the stress response in mental disorders. Biological markers include the study of DNA, RNA and proteins (extraction from blood sampling) as well as the performance in functional MRI (fMRI), psychophysiological and neuropsychological tasks. The following assessment domains are covered in BeCOME and described in detail below:

#### Omics

In this study, we plan to include a number of omics-based biomarkers from peripheral blood for patient stratification and grouping. The possible levels of investigation include genetics, epigenetic measures with DNA methylation, non-coding RNAs but also other epigenetics marks such as histone modifications as well as proteomics, gene expression and metabolomics. For these measures, we carry out blood draws at baseline as well as at follow-up visits that include EDTA blood for DNA extraction (genome-wide genotyping and DNA methylation), Pax-Gene RNA tubes for messenger RNA (mRNA) expression as well as small non-coding RNAs, serum and plasma for proteomics and metabolomics. Plasma can also be used to assess microRNAs circulating in exosomes. Finally, only at the baseline visit, we collect peripheral blood mononuclear cells using Biocoll separation. At least 30 Mio. cells are stored for each individual and these can be used as a source tissue for induced pluripotent stem cell programming as well as functional assays in live mononuclear cells. Cells are stored with a protective medium containing 10% dimethyl sulfoxide (DMSO) at cryogenic temperatures (storage below − 130 °C) in the gas phase of nitrogen.

All samples are stored in our biobanking unit (http://www.psych.mpg.de/1495662/bioprep). As omics assays evolve rapidly, the exact method for each of the assessments will only be selected once assays can be run collectively in several hundred individuals. Processing large number of samples together will reduce batch effects.

#### Neuroimaging

**Fig. 2:**
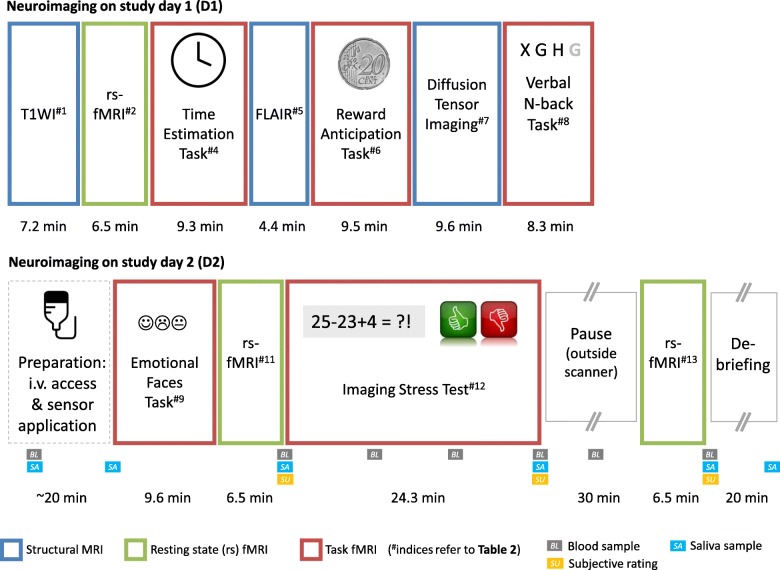
Overview of neuroimaging procedures in BeCOME. Legend: ^#^indices refer to Table 2

The acquisition of neuroimaging data in the BeCOME study is organized in two sessions that take place on D1 and D2 in a 3 Tesla MRI scanner (Discovery MR750, General Electric, Milwaukee, U.S.A.) using a 32-channel coil (see Fig. 2 for overview of the MRI procedures and Table 2 for sequence details). Both MRI sessions begin at defined times to minimize circadian influences. For paradigms that require active participation, explanations and instructions are given by experienced MR technicians, including a training session on a personal computer outside the MRI scanner. Instructions are standardized, but may be varied and expanded to guarantee that the paradigm and task has been understood. For the MRI session, all paradigms were programmed and delivered using Presentation (Neurobehavioral Systems, Berkeley, USA), displayed an MRI-compatible 30″ LCD (OptoStim, 1680 × 1050 pixels, 250 Hz, medical research GmbH, Cologne, Germany), and combined with a Lumina response box (LS-Line, Cedrus, San Pedro, U.S.A.). Participants wear noise cancellation headphones and ear plugs during the whole MRI session (Optoacoustics, Moshav Mazor, Israel).

**Table 2:** MRI sequence parameters and additional recordings

Paradigm Measurement	Sequence details	Additional recordings
**Day 1** #1 High resolution T1-weighted image^LOC^	Sagittal FSPGR 3D BRAVO, TE 2.3 ms, TR 6.2 ms, TI 450 ms, FA 12°, FOV 25.6 × 25.6 × 20.0 cm^3^, matrix 256 × 256 × 200, FDir S/I (7.2 min)	None
#2 Resting state functional MRI (rs-fMRI)	2D Gradient Echo EPI, oblique, AC-PC alignment, FA 90°, TE 30.0 ms, TR 2.5 s, interleaved/ bottom-up, no dummy scans, FOV 24.0 × 24.0 cm^2^, ST 3.0 mm, SP 0.5 mm, 42 slices, matrix 96 × 96, FDir R/L, acceleration factor 2, 155 volumes (6.5 min)	Eyetracking/ pupillo-metry, respiration belt, PPG
#3 EPI image with high tissue contrast	2D Spin Echo EPI, same geometry as #2, TE 37.6 ms, TR 10 s, FOV 24.0 × 24.0 cm^2^, ST 2.5 mm, SP 0.5 mm, matrix 96 × 96, FDir R/L, acceleration factor 2, no dummy scans, 2 volumes (0.7 min)	none
#4 Time estimation task	As #2, 224 volumes (9.3 min)	Eyetracking/ pupillo-metry,respiration belt, PPG
#5 FLAIR	2D axial Fluid Attenuated Inversion Recovery, TE 160 ms, TR 11 s, TI 2250 ms, refocusing FA 111°, FOV 22.0 × 22.0 cm^2^, ST 4.0 mm, SP 0.8 mm, matrix 352 × 224, FDir A/P (4.4 min)	none
#6 Reward anticipation task	As #2, 230 volumes (9.5 min)	Eyetracking/ pupillometry, respiration belt, PPG
#7 Whole brain Diffusion Tensor Imaging (DTI)	2D Spin Echo DTI, TE 60.9 ms, TR 8 s, interleaved/ bottom-up, 66 diffusion directions, 5 initial B0 images, FOV 25.6 × 25.6 cm^2^, ST 2.0 mm, SP 0 mm, 60 slices, matrix 128 × 128, Fat Saturation B0 images repeated using an inverted phase sampling for later unwarping of geometric distortions (9.6 min)	none
#8 Verbal n-back task	As #2, 200 volumes (8.3 min)	Eyetracking/ pupillo-metry, respiration belt, PPG
**Day 2** #9 Face matching task^LOC^	As #2, 230 volumes (9.6 min)	Eyetracking/ pupillometry
#10 EPI image with high tissue contrast	As #3 (0.7 min)	none
#11 rs-fMRI	As #2 (6.5 min)	Eyetracking/ pupillometry
#12 Imaging stress test (IST)	Gradient Echo, oblique, AC-PC alignment, FA 90°, TE 20.0 ms, TR 2.0 s, interleaved/bottom-up, no dummy images, FOV 24.0 × 24.0 cm^2^, ST 3.0 mm, SP 0.5 mm, 40 slices, matrix 96 × 96, FDir R/L, acceleration factor 2, 760 volumes (25.3 min)	Skin conductance level, pulse, PPG, ECG, repeated blood samples
#13 rs-fMRI^LOC^	As #2 (6.5 min)	none

*Abbreviations: AC-PC* anterior/posterior commissure, *A/P* anterior/posterior, *ECG* electrocardiogram, *EPI* echo planar imaging, *FA* flip angle, *FDir* frequency encoding direction, *FOV* field of view, *IR* inversion recovery, *LOC* sequence preceded by 3-plane localizer, including new pre-adjustments, *min* minutes, *PPG* pulse plethysmography, *R/L* right/left, *S/I* superior/inferior, *SP* slice spacing, *ST* slice thickness, *TE* time of echo, *TI* inversion recovery time, *TR* time of repetition; (duration in minutes)

The course of the MRI measurements on D1 (total average time spent in the scanner on day 1: ~ 70 min) is characterized by alternating functional and anatomical acquisitions, in order to grant pauses in-between functional scans and to minimize task carry-over effects. All day 1 fMRI measurements are accompanied by eye-tracking/pupillometry (EyeLink 1000 Plus, SR Research, Ottawa, Canada) (250 Hz sampling rate) for which a short calibration session is required after the participant’s positioning in the scanner. In addition, respiration and heart rate are monitored using GE’s respiration belt and plethysmography. Shortly before tasks are started, participants are verbally reminded of the instructions through the headphones. Sequences of day 1 are as follows:

- High resolution T1-weighted image: This sequence serves as a main anatomical reference and as basis for morphometric studies.
- Resting state functional MRI (rs-fMRI) using a whole brain echo planar imaging (EPI) sequence over 6:28 min, with a black fixation cross against a light-grey screen. The instruction for this task is: “Please lie as still as possible and fixate on the crosshairs. Try not to fall asleep. Eye-blinking is allowed.”
- High contrast single spin-echo planar imaging to support optimal spatial post-processing of functional time series.
- Time estimation task: This fMRI task focuses on processing of positive, negative and ambiguous feedback [19, 20]. It requires the participant to repeatedly estimate a time span of one second, starting when a fixation cross disappears, and then to press a response button. During this paradigm a total of 84 stimuli is presented in about 9:20 min, depending on the participant’s performance. The participant’s response triggers the immediate presentation of one of three graphical feedback symbols which are isoluminant and shown for 1500 ms: The feedback comprises either a green tick for ‘correct’, indicating sufficiently accurate approximation of one second inside the target time window, or a red cross for ‘incorrect’, indicating an answer outside the target window, or a black question mark for ‘uncertain’, concealing a clear feedback. The uncertain feedback is given in 30% of the trials. After each trial, the duration of the target time window is adapted to achieve a balanced proportion of successful and unsuccessful trials. The intertrial interval varies between 1800 and 2000 ms during which an empty screen is shown. The fixation cross is shown for 400 to 600 ms.
- Axial FLAIR sequence is acquired to support the screening for incidental findings and to allow for the segmentation of WM lesions.
- Reward anticipation task based on a monetary incentive delay task [21]. Again, stimuli, which are isoluminant to the background, are used to avoid the light reflex for pupillometric readouts. One trial consists of the projection of one of three graphical, abstract symbols for 6 s. These symbols announce three different types of trials: type 1, a trial that allows to win a small amount of money; type 2, a trial with verbal feedback on the performance but no monetary incentive; type 3, a trial with no response requested. After the two reward conditions (type 1 and 2), the symbols are followed by a short white screen flash (100 ms) to which the participant should react as fast as possible by pressing a button. After another 1000 ms, a symbol indicating either win of money (€), fast performance (✓) or too slow response (X) is shown for 1500 ms, followed by an update of the gain balance shown for 2000 ms. An adaptive algorithm adjusting the allowed response time window ensures that participants will succeed in approximately 50% of their responses across the session. Intertrial intervals vary between 3000 and 6000 ms during which a fixation cross is shown. For a full description of our adaption of the original task by Knutson et al. [21], see Schneider et al. [22].
- Whole brain diffusion tensor imaging (DTI) and auxiliary files to allow for distortion correction procedures are acquired as basis for microstructural analyses.
- Verbal n-back task: This task reliably elicits working memory circuits in neuroimaging studies [23, 24]. A total of 8 randomized blocks (each comprising 16 stimuli over 40 s) of the type 0-back, 1-back, 2-back and fixation are displayed. Before each block, the respective instruction is displayed for 6 s. Stimuli are small and capital letters displayed for 500 ms with an additional 1000 ms during which answers are collected. A pause of 1 s is interposed before the next stimulus.

On day 2, participants perform two further functional tasks, again after specific instructions and preparations. Saliva is sampled upon arrival at the MR scanner and repeatedly during the second paradigm to assess the endocrine stress response. In addition, a peripheral venous cannula is set up in a subset of participants to repeatedly sample blood during the second paradigm. After positioning on the MRI table, magnetic resonance compatible sensors for electrocardiogram (Ag/AgCl multitrodes, EasyCap, Herrsching, Germany), pulse plethysmography (Nonin 8604D pulse oximeter, Nonin Medical Inc., Plymouth MN, USA) and SCL (two Ag/AgCl electrodes; left index and middle finger; Brain Products GmbH, Gilching, Germany) are attached and calibrated in order to record autonomous nervous system signals (sampled and stored using the BrainVision ExG AUX Box, BrainVision ExG MR Amplifier, and BrainVision Recorder software 1.0, Brain Products GmbH, Gilching, Germany). Of note, the instructions for the second paradigm, a psychosocial stress test, only contains vague information that the task is to perform mental arithmetic while being monitored and evaluated. Details on the aversive feedback elements are not communicated prior to the experiment. In consequence, a detailed debriefing is performed after the entire experiment during which the false and knowingly aversive elements of the feedback (mainly under-average performance) are uncovered. Functional tasks of day 2:

- Face matching task: This task is an adaptation of the paradigm originally presented by Hariri et al. [25] that is widely used to study emotional face processing [26]. We implemented a version with a mixed block/event-related design, consisting of 8 blocks over a total of 9:35 min, during which either faces need to be matched according to their emotional expression, or, as a control condition, simple geometrical objects need to be matched according to their shape. As face stimuli we use examples from the Ekman faces collection [27] with neutral, fearful, sad, angry and happy facial expressions, adjusted graphically in terms of size, average luminosity, and with hairstyle or jewelry being masked.
- Imaging stress test (IST): This paradigm is an adaptation of the Montreal IST [28, 29]. Before the actual stress experiment, a 6:28 min baseline resting state fMRI is collected. The stress experiment includes three fixed phases of 8 min each referred to as *pre-stress*, *stress* and *post-stress* phase. During each phase, 5 blocks of arithmetic tasks (each 55 s) followed by 45 s of fixation cross are presented. The main instruction is to solve arithmetic problems (including simple additions and multiplications, presented for ~ 4 s [adaptive]) as fast and accurate as possible and to select the correct answer (a number between 0 and 9 on a dial) by using the response box. During the stress phase, psychosocial stress is exerted by aversive feedback (announcements of being watched and monitored; screen elements indicating ostensible under-average performance; repeated, scripted negative verbal feedback). Following the submission of an answer, a mathematically true feedback is printed on the screen after 1.5–3 s, before the next calculus appears. After the 24-min stress experiment, a 30-min recovery phase is appended, with the participant resting on the MRI table outside of the magnet, but autonomous nervous system recordings are continued. Eventually, the participant is positioned back inside the MRI scanner and second resting state fMRI of 6:28 min is acquired. After completing the IST, all devices are removed and the participant is debriefed as explained above. Figure 2 details the time points of parallel blood or saliva collections and subjective ratings.

Anatomical images (sagittal T1-weighted images, axial FLAIR images) are screened by an experienced MRI reader and verified by a board certified radiologist. Standard operation procedures exist how participants and their general practitioners are informed about incidental findings. To meet ethical standards, the general threshold for informing participants is kept low. Only a small percentage of cases with incidental findings (currently< 10%) are excluded from the study post-hoc, e. g. cases with distinct hints towards a neurodegenerative disorder.

#### Psychophysiology: tasks and procedures

The psychophysiological measurements occur on 2 days. On D1 (~ 10 AM), after the set-up with electrodes and sensors, participants undergo a habituation session. With the simple instruction that this session is a brief habituation session, a startling noise is presented four times and three visual stimuli (geometric forms) are each presented three times. In the fear conditioning session, two conditioned stimuli are followed by aversive, unconditioned stimuli (US) during conditioning (CS+, 75% reinforcement schedule), whereas a safety stimulus (CS–) is not followed by an US. The US follows at stimulus offset and comprise either an electrical shock to the back of the right wrist or an air puff to the larynx [30], dependent on the preceding CS+. Participants receive the following instruction: “The geometric forms may be followed by mild electrical shocks or air puffs”. During the fear extinction session that immediately follows, the CS+ paired with the electrical shocks is presented without any following shocks, interspersed with CS–. On day 2 (~ 9 AM), all three stimuli are presented again in the recall session: the safety stimulus, the extinguished stimulus and the un-extinguished stimulus (CS+ followed by air puffs); again neither electrical shocks nor air puffs are administered during this run, as its sole focus is on memory recall (of the extinction, fear and safety memory). Participants will receive the same instruction as on the first test day. After the recall session, one unsignaled air puff is administered and the session is continued without any US. This allows the evaluation of any possible differences in the return of fear through reinstatement.

The stimuli consist of simple geometric shapes and are presented for 4 s each, with inter-stimulus-intervals jittered between 12 and 16 s, displayed in a pseudorandom order (≤ 2 consecutive presentations of the same stimulus). Seventy-five percent of trials contain an auditory startle probe at 3.0 or 3.5 s, (104 dB white noise delivered via head phones), whereas behavioral ratings occur before, during and after the psychophysiology recordings on day 1 and day 2. One US consists of electrical shocks which are pulses of 20 ms duration with typical intensities between 3 and 25 mA, generated by a Digitimer Stimulator (Model DS7, Digitimer Ltd., Hertfordshire, United Kingdom). Stimulation intensity is individually titrated before the psychophysiology measurement following a staircase protocol. The initial shock intensity is set at 0.5 mA and 0.5 mA increments are used to find the level at which shocks will be uncomfortable but not painful. The other US is a 9 bar airblast of 250 ms duration (see [30]) delivered to the larynx from a distance of approximately 1–2 cm.

#### Psychophysiology: Read-outs

Skin conductance level and responses (SCR) and heart rate (variability) are assessed with the wireless Electrodermal Activity and Pulse Plethysmogram BioNomadix module with Biopac’s MP150 system. SCR electrodes are placed on the palm of the left hand, the pulse plethysmogram sensor is placed on the left thumb. Participants enter the behavioral ratings to stimuli on a computer keyboard with their right hand.

Eyeblink electromyographic (EMG) responses to startle sounds are measured by two electrodes on the skin surface overlaying the left orbicularis oculi muscle, after thorough preparation of the surface of the skin. A ground electrode is placed behind the left ear. The EMG signal is conducted with a dual Wireless EMG BioNomadix Pair to the MP150 system. The software package Acknowledge 4.1 is used for recording (1000 Hz) and initial preparation of data before export to MATLAB.

Pupillometry is assessed with the Eyelink 1000Plus system via the desktop mount with head support in the blinded laboratory room (artificial light only). After optimizing the pupil detection, initial calibration and validation of eye gaze (to minimize the fixation error), data is recorded of the right eye with a sampling rate of 250 Hz. Participants are instructed to fixate on the fixation cross whenever one is present and stimuli appear small and centered on the screen to minimize differences in pupil size due to gaze direction.

#### Actigraphy

Actigraphy is measured between the screening visit and the morning of D2 (approximately 14–28 days) by ActiSleep Monitors (Actigraph, Pensacola FL) or Daqtometers (Versions 2.3 and 2.4, Daqtix, Germany), which are placed on the non-dominant wrist. Devices are set to sample acceleration every second and to store activity counts every 30s as the mean of all samples within the storage interval [31], the result of which can be compared with subjective daily sleep logs.

#### Heart rate variability (HRV) measurements

A bipolar, portable mini-electrocardiogram device (Faros 180°, Biosign GmbH, Ottenhofen, Germany) is applied after the end of the fMRI measurements on D1. Controlled recordings are obtained during resting (5 min) and deep breathing (1 min). The Valsalva and orthostasis tests are optional. The device is worn by the participant until the next morning (~for 18 h overnight). The device is removed and the data are read out on a standard PC in the morning of D2. Electrocardiogram samples have a resolution of 500 Hz, allowing for precise peak detection, and accompanying accelerometer data have a resolution of 50 Hz. Data analysis is programmed in-house in Matlab (Pan Tompkin’s algorithm) and *R*, supported by open-source packages (HRVR), and covers semi-automated artifact correction, automated peak detection and generation of basic standard heart rate variability parameters.

#### Neuropsychology: tasks and procedures

The cognitive test battery includes tests from the Test of Attentional Performance (TAP) [32], the “Materialien und Normwerte für die neuropsychologische Diagnostik” (MNND) [33], the Wechsler Adult Intelligence Scale (WAIS-IV) [34], as well as the Trail Making Test (TMT) [33], the d2-R [35], and the “Mehrfachwahl-Wortschatztest” (MWTB) [36]. Three basic domains of attention, executive functioning, and memory are assessed with the following tests:

- Episodic memory (MNND): A brief text is read out loud to the subject, who is instructed to memorize the content. The subject is required to reproduce the text in as much detail as possible. Reproduced contents (verbatim and analogous) are assessed immediately after the presentation (short delay) as well as 30 min later (long delay).
- Working memory (2-back task, TAP): A series of numbers are presented on a screen. The participant is required to respond with a button press whenever a number is the same as the number second to last. Reaction time, omission and commission errors are recorded.
- Inhibitory control (GoNogo task, TAP): In this task, the symbols “X” and “+” are presented in alternating sequence on a screen. The participant is required to respond with a button press upon appearance of “X” (Go trial), but not “+” (Nogo trial).
- Cognitive flexibility (TAP): A pair of stimuli, consisting of a number and a letter, is presented on a screen. The subject is required to respond with either a button press on the right or the left side depending on the required response. For example, for the first pair, the button needs to be pressed on the side of the letter, for the next pair on the side of the number, and so forth. Errors and reaction time are assessed.
- Word fluency (MNND): The participant is asked to produce as many words as possible beginning with the letter “S” during three minutes.
- Sensitivity to interference (Stroop, MNND): The participant is presented with three consecutive templates. The first template shows colored circles and the participant is required to name the color of each circle as fast as possible. On the second template, the participant is shown words in differently colored font and is asked to name the color of each word as fast as possible. On the third template, colored words (e.g., “blue” or “green”) are presented to the participant in differently colored fonts. Again, the participant is required to name the color of the word as fast as possible. Processing time, Stroop mistakes (reading the color word on the third template) and other mistakes are assessed.
- Attention/flexibility (TMT): In this test, 25 circles are distributed over a sheet of paper. In the first part (TMT-A), the circles are numbered from 1 to 25 and the participant is required to draw lines to connect the numbers in ascending order. In the second part (TMT-B), the circles include both numbers and letters and the subject is required to connect the circles in alternating ascending order (1-A-2-B etc.). Processing time for TMT-A and TMT-B as well as the ratio (TMT-B/A) are registered.
- Attention (d2-R): This is a measure to assess the ability to concentrate. The participant is presented with 14 lines (each 20 s) of characters, of which one character (the letter “d” with two dashes) needs to be marked. All other characters (b, q, p) or the letter “d” associated with one or three dashes need to be omitted. Concentration ability is calculated from the number of processed items and processing speed.
- Crystallized intelligence (MWTB): The MWTB is a choice vocabulary test of verbal intelligence, developed to match the construct of crystallized intelligence. Participants have to indicate the real word in a row of five words. The number of correctly answered items (37 in total) provides an estimate of crystallized intelligence.

The neuropsychological test battery is administered by trained research personnel (Psychology students and medical technical assistants). The training consists of observations of five test sessions and being observed in five test session by trained research personnel. Each assistant is then observed at least once by a trained neuropsychologist before being admitted as assessor of neuropsychology data.

#### Probabilistic social reward learning task

In order to assess implicit social learning and cue integration, we employ an established probabilistic social reward learning task [37]. In this computational modeling task, participants decide between one of two cards with varying winning probabilities. At the center of the screen, a face of a computer-generated avatar is presented. At the beginning of each trial, the face looks towards one of the two cards. The probability of the gaze providing a helpful advice is systematically manipulated independent of the changing winning probabilities of the cards. After the saccade, the participant is asked to choose a card and wait for the feedback in which the outcome (correct/wrong) is presented. Both cards are associated with reward values. When a choice was correct, the reward value of the chosen card is added onto a cumulative score, which is updated in the feedback phase. In the instruction, the participants are informed that the winning probabilities of each card would change during the experiment. However, no explicit information is given with respect to the social cue. Behavioral responses of this task will be modelled using hierarchical generative models [38] in order to estimate the hidden states that govern the learning process about the card and gaze probabilities, respectively. Applying the model also allows to individually estimate the extent to which participants are integrating the social information during their decision making process. After the screening visit, participants receive an online link in order to get to an internet-based version of the paradigm, which was implemented in PsyToolkit [39, 40] (https://www.psytoolkit.org). This task consists of 120 successive trials followed by a brief questionnaire to measure the subjective exploitation of social and non-social information.

#### Self-report measures: interviews, rating scales and questionnaires

#### Diagnostic Interview

A shortened and slightly modified lifetime and 12-month version of the computer-assisted Munich-Composite International Diagnostic Interview (DIA-X/M-CIDI) [41, 42] is used to assess symptoms, syndromes and diagnoses of the following mental disorders according to DSM-IV [13]: nicotine use and dependence (section B), anxiety disorders (panic attacks, panic disorder, agoraphobia, specific phobias, generalized anxiety disorder; section D), depressive episodes and dysthymia (section E), mania and bipolar disorders (section F), psychoses (section G), alcohol use/disorders (section I), obsessive-compulsive disorders (section K), illegal substance use/disorders (section L) and post-traumatic stress disorder (section N). The M-CIDI additionally collects information on onset, duration and severity of mental disorders. The M-CIDI was developed on the basis of the World Health Organization’s CIDI version 1.2 [43] to additionally cover ICD-10 criteria. Psychometric properties of the DIA-X/M-CIDI have been reported elsewhere [44–46]. The interview is conducted face-to-face by trained study assistants who are regularly supervised by a staff psychologist and undergo repeated training in the administration of the DIA-X/M-CIDI. Each interview undergoes a plausibility check according to a standard procedure.

#### Observer-administered rating scales

Past-week severity of depressive symptoms is rated with the Montgomery-Åsberg Depression Rating scale (MADRS) ([47], German version: [48]). In order to ensure interrater reliability, raters use a structured interview guide ([49], own translation into German) for gathering the information needed for coding the MADRS.

The Panic and Agoraphobia Scale (PAS) by Bandelow [50] is administered to collect information on past-week occurrence of panic attacks, agoraphobic avoidance, anticipatory anxiety, disability due to panic and agoraphobia and functional avoidance. Each assessor is receiving training in the administration of the PAS scale.

In order to continuously ensure the quality of data from observer-based ratings, raters undergo regular training sessions and are supervised by a staff psychologist. Before being allowed to conduct the first interview by themselves, trainees are required to observe an experienced rater administering the interview and to conduct an interview with an experienced rater sitting in the session. In both situations, their coded sum score needs to fall within 2 points of the total score of the experienced rater. The training is continued until the criterion is met. Questions and difficulties arising from the application of the instruments are addressed in weekly meetings or immediately by contacting the supervisor. Deviations from the rating protocol are checked in regular interrater reliability meetings and booster training sessions.

#### Questionnaires

- Socioeconomic status (SES): Education, occupation, current employment status, household composition/income and social class status of participants and their spouses is assessed with a questionnaire that we developed on the basis of the recommendations by the German Statistical Federal Office [51]. It includes a section for the assessment of the SES of the participant’s family of origin to determine the SES during the forming years of childhood and youth. The questionnaire additionally includes the German version of the Quality of Marriage Index (QMI-D) ([52], original version: [53]) and allows for the determination of a social prestige/status index according to international standards [54].
- The following depression- and anxiety-related symptom measures are used for a deeper dimensional characterization of participants:
  Beck-Depression-Inventory (BDI-II [55], German version: [56]) for the measurement of depressive symptom severity within the past 2 weeks.
  The State-Trait-Anxiety Inventory (STAI [57], German version: [58]) with the sum score of the state anxiety scale (A-State, form X1) representing an intensity measure of a transient emotional state characterized by tension, uneasiness, nervousness, fear of future events and arousal of the autonomic nervous system and the sum score of trait anxiety (A-Trait, form X2) indicating a general anxiety predisposition that is stable over time.
  The Body Sensations Questionnaire (BSQ) and the Agoraphobic Cognitions Questionnaire (ACQ) developed by Chambless et al. [59] for the assessment of the fear of fear (German version: [60]). The ACQ comprises three subscales: agoraphobic cognitions, loss of control and physical concerns.
  The Intolerance-of-Uncertainty Scale (IUS) ([61], German version: [62]) assesses reactions to ambiguous situations, uncertainty and future events and is listed in the RDoC matrix as a self-report measure for potential threat.
- Personality measures: The Tridimensional Personality Questionnaire (TPQ) ([63], German version: [64]) assessing novelty seeking, harm avoidance, reward dependence.

The Behavioral Inhibition and Behavioral Approach System (BIS/BAS) scales to assess approach motivation and individual differences in the sensitivity to reward and punishment ([65], original version: [66]). The newest version of the RDoC matrix lists BIS also as self-report measure for the RDoC domain potential threat.

A four-item questionnaire (IE-4) is used to measure locus of control (LOC) [67], the personal belief about whether life is controllable by own actions (internal LOC) or by external factors outside one’s influence (external LOC). LOC is assessed because it might have a strong influence on reward learning and valuation.

For a categorical and dimensional self-assessment of DSM-IV personality disorders, we use the “Assessment of DSM-IV Personality Disorders” (ADP-IV) by Schotte et al. ([68], German version: [69]). The ADP-IV specifically asks for personality features that cause stress, problems and social conflicts.

(d) Aspects of social processes are measured with the following questionnaires:

The Relationship Scales Questionnaire (RSQ) ([70], German version: [71]) was chosen for the assessment of attachment style because it operationalizes several theoretical concepts of attachment theory and it can be completed by singles. The RSQ allows for the dimensional assessment of four attachment prototypes (secure, preoccupied, dismissing-avoidant, fearful-avoidant) according to the four-category model of Bartholomew and Horowitz [72]. The RSQ comprises the 18 items of the Adult Attachment Scale (AAS [73]) from which the three subscales closeness, dependency and anxiety in relationships can be derived. The RSQ can also be scored in regard to the two dimensions attachment-related avoidance and attachment-related anxiety [74, 75].

Interpersonal pleasure is measured with the German Translation of the Anticipatory and Consummatory Interpersonal Pleasure Scale (ACIPS) [Skala der erwarteten und vollendeten zwischenmenschlichen Freude, translated by K. Kirst, University of Wisconsin-Madison] [76]. Four factors (general social interactions, close relationships, shared interests and experiences as well as family-related interactions) can be derived from the ACIPS [77].

The size of the social network is estimated with the Social Network Questionnaire (SNQ) [78] that has been derived from the social contact circle interview [79]. The SNQ asks participants to list the names of all individuals (household members, family members, friends, colleagues, neighbors, others) with whom they were in contact within the past 4 weeks.

Empathy Quotient (EQ) and Autism Quotient (AQ) are measured with the questionnaires originally developed by Baron-Cohen et al. (EQ: [80], German translation: [81], AQ: [82], German translation: [83]).

(e) Exposure to trauma, critical life events and coping strategies: The German version of the childhood trauma questionnaire (CTQ) [84] by Wingenfeld et al. [85] is used to assess sexual, physical and emotional abuse as well as physical and emotional neglect experiences during childhood. The CTQ is listed as a self-report measure for the RDoC domain “sustained threat” [86]. For collecting more detailed information on traumatization during childhood and adolescence (up to age 18) in terms of exposure frequency during certain age periods and past/current impact of the event, we additionally administer the short inventory for the assessment of early traumatic life events (K-IFTL). The K-IFTL is a modified version of the Early Trauma Inventory (ETI [87], German version: [88]) and covers the domains physical punishment/assaults, unwanted sexual experiences and general traumas (e.g., death of a close friend). We additionally added a section on emotional traumas to the K-IFTL, as emotional traumas are the most frequent childhood traumas in our patient population. Lifetime exposure to accidents, natural disasters, criminal acts, physical assaults and sexual traumas together with information on age at traumatization and exposure duration is assessed within the post-traumatic stress disorder (PTSD) section of the DIA-X/M-CIDI [41, 42]. The trauma list N1 was modified to include occurrence frequency, duration age at exposure for each trauma category. Information on current (past 6 months) exposure to negative life events is collected with a modified version of the Munich event list (MEL by [89], described in detail in [90]). Since the RDoC domain “loss” was not completely covered by our environmental measures but is of importance for the development of depressive and anxiety disorders, we developed a questionnaire for assessing experiences of loss over lifetime. This self-developed loss event questionnaire (LEQ) assesses the frequency, past/current impact and age at occurrence of several loss events such as death of parents, close attachment figures, siblings, spouses, children, close friends, separation/divorce of parents, separation/divorce from spouse/partner as well as severe (life-threatening) illnesses of loved ones.

The following stress coping strategies are assessed with a validated German stress coping inventory (Stressverarbeitungsfragebogen-78 item version, SVF78, [91]): Play down, guilt denial, substitutional satisfaction, situation control, reaction control, positive self-instruction, need for social support, active avoidance, flight tendency, rumination, resignation, self-accusation. The SVF78 additionally allows for the differentiation between stress-reducing (positive) and stress-augmenting (negative) strategies. For the measurement of psychosocial stress-resistance we used the 11 item version of the resilience scale (RS-11) ([92], German version: [93]). The RS-11 is a one-dimensional scale that conceptualizes resilience as a protective personality factor.

#### Data acquisition

Each questionnaire (except for the loss event and the SES questionnaire) was computerized and collected with an online survey tool (collector 2015.Q2 by survalyzer). The battery of questionnaires is split into three packages to reduce the load associated with filling in the questionnaires. For each package, participants receive a separate online link after the screening visit.

#### Repeated measurements

In MPIP patients, the MADRS, BDI-II, PAS and STAI-X1 are repeatedly assessed at each in-house study visit (days 14, 28 and 56) to assess the course of symptoms over time.

#### Data management

A biomedical research portal (software CentraXX by Kairos, Germany) will be used for the secure storage and management of data. The software includes a biobanking and a clinical trial management system supporting a quality-assured execution of the study. Data are entered in eCRFs (electronic case report forms) with automatic checks whether values fall within the valid range. Incomplete eCRFs are flagged. Every eCRF is checked by a second data entry assistant in regard to transferring errors and inconsistencies. Only complete and quality controlled data (flagged green) are released.

Data confidentiality and security are enforced through several mechanisms. Access to the database is password secured and restricted by role-based rights. The visibility of confidential data and the type of activity an individual user is allowed to undertake is regulated by his/her role in the study and privileges associated with the personal login data. The security architecture of the research portal implements German data protection laws.

The principal investigator (EBB) and coordinators of the study (TB, VS, PS, SL, AE) have full access to the dataset. Access to the data for research purposes is granted by the PI upon request. Topics for publication need to be approved by the PI and communicated with the coordination team who oversee authorship regulations. Study participants, who signed that they want to be informed about publications arising from the study, receive an email notification when a publication appears. For data protection reasons, the full data set including omics data cannot be made publicly available. Access to data of published results will be granted upon request.

### Statistical analysis

The overall aim of the study is to integrate multi-level information across all assessment domains and to yield biology-based subclasses of mental disorders by using cluster analytical techniques. Previous attempts to identify biomarkers and biotypes of mental disorders have failed to identify reproducible biomarkers. So far, these studies have included fewer assessment domains, such as only neuroimaging, neurotransmitter measurements or self-report data (see e.g. [94–96]). Given the complexity of mental disorders that associate with domains like personality, life history as well as environmental, behavioral, neurobiological, and genetic factors, it is reasonable to assume that heterogeneity in mental disorders can be best understood by considering as wide a range of modalities and data sources as possible. The comparative utility of different data modalities has been examined in studies seeking to predict disease outcomes (e.g. [97]), but has not been adequately addressed with regard to more general biomarker discovery for mental disorders. To ensure that the rich data collected in this study will be used in the most beneficial way, the core analyses of this study will be planned and carried out in alignment with expert recommendations regarding development of biomarkers for psychiatric disorders [98, 99].

Two of the primary concerns that need to be addressed in our analysis approach are the integration of information from multiple data sources and the ratio of observations to possible variables to be examined. To this end, the core assessment domains - omics, neuroimaging, psychophysiology, neuropsychology, and self-report measures – will first be analyzed separately to reduce the dimensionality of the feature space. These partial analyses will include feature extraction (e.g. PCA) and feature selection steps that will inform the input into a multi-modal unsupervised learning analysis. For example, data-driven approaches will be combined with expert-based feature selection (e.g. evidence from the literature, meta-analyses) in order to include domain knowledge in the selection process. The goal of these dimensionality reduction steps is to reduce the number of input variables from multiple assessment domains to a small set of validated and generalizable features. These reduced feature set will be used as input into an unsupervised learning pipeline incorporating subsampling and cross-validation steps to guard against overfitting. An appropriate clustering approach will then be chosen based on the outcome from the previous data integration steps. Depending on the dimensionality of the final feature set, ensemble learning will be considered as a possibility for separating the contribution of the different assessment domains. This may be particularly beneficial when considering that previous research has shown domains such as self-report questionnaire assessments to account for a much larger portion of variance than omics or neuroimaging data in explaining behavioral or disease phenotypes (e.g. in alcohol use: [97, 100, 101]). By constructing separate models for different data domains, a “drowning out” of weaker effects that nevertheless contribute unique variance to a model may be counteracted.

While the prior reduction of the feature space is an important step in reducing the risk of overfitting, the suitability of our sample size for recovering existing effects is nevertheless of concern [102]. Thus far, no results from comparable studies examining affective or anxiety disorders have been reported, making it difficult to estimate possible effect sizes and determine case numbers accordingly. Therefore the determination of the case number was guided by studies with a comparable research agenda (e.g. B-SNIP study [103]). We plan to include at least 1000 patients or individuals affected by a mental disorder and 500 individuals without a mental disorder according to the DIA-X/M-CIDI. These sample sizes are comparable to the case numbers Clementz et al. used for the identification of psychosis biotypes [104] and are also informed by examinations of the changes in retrievable effects in prediction studies with low signal-to-noise data and varying sample and feature set sizes [105]. The feature reduction steps are used to keep the feature-to-observation ratio in a balance. Since the partial analyses and other projects resulting from data collected in this study will precede our main analysis, we will track the participants included in each prior examination of the data and use this information to partition the dataset into a training set including all participants previously examined and a test set including only those participants untouched in prior analyses. Consequently, any models developed based on data collected in this study will be tested on a held-over sample to avoid overfitting due to in-sample model validation [106].

## Discussion

Only few studies exist that have tried to identify biological subtypes of affective and anxiety disorders. Most of these studies were limited by relatively small sample sizes and a more narrow focus on only one specific biological factor (for a review see [94]). The BeCOME study tries to overcome the shortcomings of previous subtyping research by

- the recruitment of a much larger sample of patients as well as controls;
- using a multilevel assessment and in depth-phenotyping approach (including a broad range of omics, neuroimaging, psychophysiological, cognitive and self-report data)
- and by the application of experimental paradigms for the induction of responses in basic behavioral, emotional or motivational systems known to be disturbed in affective and anxiety disorders.

The activation of disorder-related systems allows for the collection of dynamic data which will carry more information about pathophysiological processes underlying the disorder than a baseline only characterization. Table 3 illustrates how the instruments and measures of the BeCOME study have been selected to characterize basic domains of functioning aligned with RDoC domains and constructs that are relevant for the psychopathology of stress-related mental disorders. The BeCOME study was explicitly designed as a multilevel assessment and deep-phenotyping study which resulted in an extensive and time-intensive research project. Participants need to invest approximately 16 h of their time for completing all study assessments and need to be free on two consecutive weekdays. These high time demands and the strict inclusion criteria likely leads to a selection bias which limits the generalizability of results. Another challenge of the study is the integration and reduction of data across multiple assessment levels. Below, we first discuss how each level of assessment can contribute to this task and then give examples for how different measures map to RDoC-like construct and the horizontal integration of RDoC constructs across tasks and measurement levels.

**Table 3:** Mapping of BeCOME assessments onto RDoC

RDoC Domain	RDoC Constructs	Genes	Mole-cules	Cells	Circuits^a^	Physiology	Behavior	Self-Report	Paradigms/Tasks
NegativeValence	Acute threat (“fear”)	Genome- and systemwide OMICS assessments (blood draws): - DNA, polygenic risk scores - Epigenetic markers (e.g. DNA methylation, non-coding RNAs, histone modifications - Gene expression - Proteomics and Metabolomics - Peripheral blood mononuclear cells (PBMC), induced pluripotent stem cells (IPSC) - In vitro dexamethasone stimulation of cells			Autonomic nervous system, amygdala, hippocampus, central nucleus, dmPFC (pl), dorsal ACC, dPAG, hypothalamus, ICMs, insular cortex, latPFC/insula, LC, OFC, PAG, pons, rostral/vent ACC	Blood pressure, heart rate, startle EMG, pupillometry, skin conductance	Estimation of the probability of aversive event (electrical shock), number of mistakes on mental arithmetic task	Symptomatic assessment of phobias (M-CID/DIAX)	Fear conditioning and extinction^b^, imaging stress test^c^
	Potential threat (“anxiety”)				Bed nucleus of stria terminalis	Potentiated startle, average cortisol levels		Intolerance of Uncertainty Scale, Behavioral Inhibition Scale (BIS), Body Sensation Questionnaire (BSQ), Agoraphobic Cognitions Questionnaire (ACQ)	Startling noise presentation^b^
	Sustained threat				Attention/vigilance network, dysregulation of amygdala/cingulate reactivity, habit systems (Striatum/ caudate/accumbens), hypothalamic nuclei, PVT	Dysregulated HPA axis	Anhedonia measures, decreased appetitive behavior (MADRS item No. 5), memory retrieval deficits (episodic memory task)	Childhood trauma Questionnaire (CTQ), KIFTL = modified version of the early trauma inventory, section N of M-CIDI/DIAX	Episodic memory task^d^
	Loss				Amygdala, default mode network, dorsolateral prefrontal cortex, habit systems (Striatum/ caudate/ accumbens), hippocampus, insula, orbitofrontal/parietal cortex, posterior cingulate gyrus, PVN, reward circuitry, vmPFC	ANS, HPA axis, neuroimmune, prolonged psycho-physiological reactivity,	Sadness (MADRS item No. 1) Guilt (MADRS item No. 9), executive function, attentional bias to negative valenced information (identification of emotional face expression, response time)	Loss events questionnaire, Munich Event List	
	Frustrative nonreward				Amygdala, hypothalamus, LC, OFC, PAC, parasympathetic system, septum, striatum		Aggressive behaviors (can be derived from personality measures)		
Positive Valence	Reward responsiveness				Anterior insula, dorsal ACC, lateral hypothalamus. Medial OFC, nucleus accumbens, vental pallidum, ventromedial PFC, VTA	Pupillometry, heart rate	Response time, decision-making in social Bayes task	Behavioral inhibition system, Behavioral approach system (BIS/BAS)	Reward anticipation task^c^ (= monetary incentive delay task), time estimation task^c^ (= simple guessing task)
	Reward learning				Amygdala, basal ganglia, dorsal ACC, lateral habenula, orbitofrontal cortex rostral medial tegmentum, substantia nigra/VTA, ventral striatum	Cortical slow waves, heart rate change, skin conductance			Probabilistic social reward learning task
Cognitive Systems	Attention				Basal forebrain limbic system, dorsal/ventral attention network		Processing speed, number of mistakes		d2-R^d^, trail making test^d^
	Declarative memory				Hippocampal circuitry, PFC and PPC interactions with multiple association cortices		Short and delayed recall		Episodic memory task^d^
	Cognitive control				Frontopolar/anterior LPFC (BA10), Inhibition of DMN, dorsolateral prefrontal cortex, posterior parietal cortex, VLPFC		Response time, sensitivity to interference, inhibition		Stroop^d^
	Working memory				Dorsal parietal, dorsolateral prefrontal cortex, inferior parietal cortex, PFC-parietal-cingulate-dorsal thalamus-dorsal striatum, MD, VA thalamus, VLPFC	Pupillometry, heart rate	Number of mistakes		Verbal N-back^c^, 2-back^d^
Arousal and Regulatory Systems	Arousal				Basal forebrain nuclei to cortical circuits, cholinergic and monoaminergic nuclei projections to thalamic and cortical, Cortical circuits such as fronto-insular and dorsal anterior cingulate etc.	Heart rate, pupil size, blood pressure, pulse, reduced habituation in standard psychophysiological measures (startel respone, skin conductance), HPA axis	Startle response	Subjective ratings during stress test	Imaging stress test^c^, Fear conditioning/extinction^b^, resting state fMRI, Heart rate variability (mini-ECG), pupillometry, electrodermal activity
	Circadian rhythms Sleep-wakefulness					Pulse, heart rate	Circadian rest/activity rhythms in the real world, motor behaviors during sleep	Subjective daily sleep logs during actigraphy	Actigraphy, Mini-ECG, sleep-dependent memory consolidation (fear extinction)^b^
Social Processes	Affiliation and attachment				Amygdala, BNST, FF gyrus, Nacc, OFC, PVN, VMPFC	Immune markers, activation of sympathetic activity, HPA axis		Attachment style (RSQ) Social anhedonia (ACIPS)	
	Social communication				Amygdala		Identification of emotions	Size of social network (SNQ)	Emotional Faces Task^c^
	Perception and understanding of self				Left inferior frontal cortex, posterior cingulate/precuneus, MPFC		Evidence that one understands ownership of one’s own body parts or action (thoughts/behaviors), hallucinations	Locus of control (IE-4, attributional style questionnaire), psychotic section of M-CIDI	
	Perception and understanding of others				MPFC, precuneus, superior temporal sulcus, temporal pole, TPJ			Emotional/autism quotient (EQ/AQ)	Emotional Faces Task^c^

^a^as listed in the RDoC matrix; ^b^task performed during psychophysiological measurements; ^c^task performed during imaging measurements; ^d^task performed during neuropsychological measurements

### Omics

We will perform genome-wide genotyping followed by genotype imputation [107] of all participants and this information can be used to determine polygenic risk scores for each individual based on large genome-wide association studies (GWAS) of common psychiatric disorders, but also medical conditions or physiological, biological or lab measures, including for example scores derived from the GWAS from large imaging studies in ENIGMA, immune parameters or body mass index. In addition, we plan to map functional genetic scores based on expression or methylation or other quantitative trait loci, as available from public databases (e.g., https://gtexportal.org/home/) or our own resources [108]. Polygenic risk scores may support novel patient stratification by testing overlapping or divergent genetic risk across current diagnostic categories. Previous studies have shown that a higher polygenic risk score for schizophrenia is associated with more mood incongruent psychotic symptoms in patients with bipolar disorder [109] or that patients with features of atypical depression have a polygenic score predictive of higher BMI [110]. Besides genetics, we also aim to use other omics data to refine patient stratification. Metabolomics in peripheral blood, both at baseline as well as with antidepressant treatment have already been shown to associate with disease severity and future treatment response in depression [111, 112]. Measure of epigenetic features, such as DNA methylation may allow assessment of features driven by both, genetic as well as environmental factors [113]. Peripheral blood microRNAs are emerging as promising biomarkers and some may correlate with the function in specific neurotransmitter systems [114] or exposure to chronic stress [115]. Methodological developments, e.g., for proteomics, will allow the assessment of these parameters at higher sensitivity, decreased cost and input requirement.

Finally, the development of machine learning tools allowing the integration of a number of biomarker measures will be necessary to fully leverage the power of these methods. Such methods are increasingly used in other areas of medicine [116] but also in psychiatry [117].

### Structural and functional neuroimaging

The structural and functional MRI assay has been composed mainly under two perspectives: First, to provide a broad coverage of brain imaging data suitable to study and deep-phenotype the depression/anxiety spectrum, and second, feasibility of the protocol, particularly in terms of its tolerability by patients. Both aspects have led to a protocol that is distributed over two sessions on two different days at baseline. Here, the first, longer session is characterized by an alternation between structural and functional measurements to allow the participant for sufficient pauses between task engagements.

The MRI assay delivers a robust basis for an extraction of macrostructural, microstructural and functional markers in the sense of a circuit-directed phenotyping [7]. It comprises state-of-the art anatomical sequences including diffusion tensor imaging for morphometric studies and to derive measures of structural connectivity (‘structural connectomics’). The task battery has been aligned to RDoC schemes in that key functional domains are covered: Brain circuits underlying *positive and negative valence* are addressed by several paradigms. First, an established face matching task [25, 26] for which a large body of literature has demonstrated relevance to affective and anxiety disorders (e.g. [118]) and that is suitable to map the amygdala-centered fear circuitry. Second, reward processing is approached by an incentive monetary delay task that focusses on reward anticipation and robustly recruits ventral striatal and salience network responses [22]. Third, neural responses to positive, negative and ambiguous feedback are reliably elicited by the time estimation task that originates in the feedback-related negativity, a known event-related potential in response to negative feedback [20]. Negative and ambiguous feedback can trigger self-related processing of the brain, and abnormal responses to performance feedback were found to predict neuroticism traits [20] and anhedonia [119]. With regard to *cognitive domains*, working memory (WM) functions can be robustly mapped using a verbal n-back paradigm [24]. WM deficits are seen under acute stress [120] and are common in depression where they tend to persist beyond acute episodes [121, 122]. WM dysfunction broadly impacts other executive functions such as planning, decision making and problem solving [23], and the elucidation of its neurobiological basis is thus important. Eventually, *arousal and stress regulatory systems* are studied explicitly by the IST during which psychosocial stress (operationalized as aversive evaluation during performance of mental arithmetic) is exerted. The IST is a multimodal advancement of the Montreal IST [28, 29], characterized by pre-stress, stress and post-stress phase to allow for studies on the deflection and recovery of the stress response system at different levels (fMRI, endocrine, autonomous nervous system, and molecular such as gene expression). Its multiple layers can be exploited for combined analyses (e.g., autonomous nervous system-informed fMRI analyses, imaging genomics) or deliver separate, complementary readouts.

Resting state fMRI, an area that has strongly expanded over the last 10 years since its entry into the neuroimaging field (see reviews [123, 124]), is obtained on D1 and D2, here before and after the IST. This allows for extracting functional connectivity (FC) measures including within-subject-stability measures and stress-induced changes. Homogenization and in part automatization of fMRI preprocessing are first steps towards a systematic data-to-information breakdown. Next, the extraction of carefully selected, task-specific, regional BOLD amplitude contrasts is needed, before aggregation with other modalities and data-driven definition of clinical subtypes can be applied. Given the rich phenotypes and depth within each domain and per subject, a large sample is needed to avoid overfitting.

### Psychophysiology

The psychophysiological tests addressing fear learning and recall will include eye blink startle EMG and SCR to relate our findings to other samples and the current body of literature. In addition to that we have incorporated pupillometry into our analyses as a sensitive readout for differential fear learning [125], and we have validated this comparatively novel readout against the state-of-the-art in an initial study on healthy participants [126]. Incidentally, this work revealed that pupil dilation closely matched subjective US expectancy ratings and did not habituate like startle EMG or SCR. Slow pupil dilations seem to more closely reflect valence-unspecific emotional arousal like SCR [127], whereas studies on startle EMG indicate that this measure captures valence-aspects of fear learning to a larger extent [128]. The recall session on D2 will allow the evaluation of fear and extinction memory consolidation, with an additional procedure of presenting two unsignaled USs to assess reinstatement of fear. With these readouts and sessions, we aim to disentangle various physiological and cognitive/affective processes, including non-associative learning (habituation), differential fear learning and uncertainty. Classical and Bayesian statistical analyses will be applied in order to optimize robust extraction of parameters for such processes at the individual level, which can then be related to the other data modalities. As an outlook, the sessions will be extended to a virtual reality (VR) setting in order to assess fear behavior in addition to physiological and subjective responses.

### Neuropsychology

Cognitive functions are known to be impaired in most patients with psychiatric disorders [129]. Cognitive deficits can precede the onset of psychiatric symptoms and often persist beyond acute episodes, indicating at least partial independence from other symptoms. For example, cognitive dysfunctions were found to persist in patients whose depressive symptoms had remitted [130–135]. Notably, however, different psychiatric diagnoses are associated with similar patterns of impairment, which underlines the lack of biological validity of current classification systems. Dysfunctions mostly occur in executive functions [135], but also arise in attention and memory functions [129]. They have been suggested to be mediated by stress-related neuropeptides [136] and shown to be associated with network dysfunctions [137–139]. However, it appears that cognitive changes can also co-vary with affective changes. For example, improvements in verbal memory, verbal fluency and psychomotor speed. Improvements in mood were most closely related to improvements in verbal memory, verbal fluency and psychomotor speed appeared most closely related to improvements in mood, whereas attention and executive function remained impaired across treatment [140]. Importantly, cognitive impairments have been shown to affect the return to work and relapse frequency.

Only few studies to date have investigated cognitive functions as possible predictors of severity of illness and therapy outcome, as well as how they could contribute to optimal therapy selection [141, 142].

Different mechanisms might be at play in younger and older populations. Late-life depression, for example, is related to possible transition to neurodegenerative disorders [143]. Furthermore, depression predicts the worsening of cognitive function in older age and better pre-treatment cognition, particularly verbal memory, is associated with better treatment response in late-life depression [144]. We aim to elucidate the role of cognitive functions, alone and in conjunction with the broad variety of other measures applied.

### Computational modeling task for assessing social reward learning

We employ a probabilistic social reward learning task, which involves learning about the changing winning probabilities of two cards. In addition, a social cue in form of a computer-generated face is presented which would, in every trial, give advice on which card to choose. We are interested in the computations that drive the learning and decision-making process for these two sources of information. The framework of predictive coding or Bayesian inference provides generative learning models of how the brain combines previously learned information with newly observed data in a Bayesian optimal manner [145]. Fitting such computational models to behavioral data acquired in our task, allows us to estimate individual approximations to Bayes-optimality, that is the estimation of subject specific parameter values that govern the learning process and extent to which participants are integrating the social information during their decision making process. Recent work in the burgeoning field of computational psychiatry has pointed to an aberrant learning process that manifests itself in error-prone inferences or conclusions when we are trying to make sense of the world. For instance, Browning, Behrens, Jocham, O'Reilly & Bishop [146] showed that trait anxiety was associated with impaired learning about environmental volatility, i.e., the change in probabilistic relationships over time. DeBerker et al. [147] applied the Hierarchical Gaussian Filters [38] to demonstrate that emotional and physiological stress reactions are tightly linked to the subjective uncertainty when learning about the probabilistic relationship between visual cues and electric shocks. Applying similar modelling approaches, we hope to gain a better understanding of the computational commonalities and or differences associated with different patient cohorts with respect to social and non-social information processing.

### Self-report measures

The selection of self-report measures for BeCOME was guided by three principles: (1) A part of the measures should cover the major domains of human behavior and functioning that have been specified by RDoC and ROAMER as useful constructs for biomedical research in psychiatry (negative/positive valence, cognitive, social processes and arousal/regulatory systems) [7, 148]. However, until today it remains unclear which questionnaires are appropriate measures that reflect specific RDoC domains [7, 148]. In 2015, when BeCOME started, only very few self-report measures had been proposed by the RDoC initiative and some of these measures were later criticized for being misplaced within the original RDoC matrix (e.g., the STAI taps into potential rather than acute threat) [149] or even removed from the RDoC matrix (e.g., BAS scales). The final report of a more recent workgroup on tasks and measures for RDoC aiming at compiling a set of standardized behavioral tasks and self-report instruments for the assessment of RDoC constructs again lacks recommendations for self-report measures [86]. For some RDoC constructs, appropriate self-report instruments simply do not exist yet. Over the course of the study, we intend to adapt psychometric measurements in BeCOME to new developments in the field.

(2) Despite the study’s focus on (neuro) biological dysregulations, a second guiding principle was that the selected instruments should still allow for a comprehensive *symptomatic* characterization of patients. We administer a structured diagnostic interview in order to be able to contrast new biological subtype findings with traditional DSM−/ICD-10 diagnoses [150]. For the discovery of new phenotypic targets for biomarker research, we intend to perform data-driven clustering and other sub-phenotyping strategies on self-report measures and integrate the findings with biological results. RDoC has already stimulated research into symptom-based alternatives to traditional DSM categories, which led to the identification of phenotypes that show stronger links with biomarker findings. For example, using a data-driven approach, Grisanzio et al. (2018) identified symptom subtypes that map onto cognitive functions, electroencephalographic and behavioral measures [151]. By referring to the RDC criteria for subtyping schizophrenic, schizoaffective and bipolar patients, Allardyce et al. [109] showed a dose-response relationship between polygenic risk for schizophrenia and the presence of mood-incongruent psychotic symptoms.

(3) Another major focus of BeCOME is the determination of lifetime exposure to traumatic and adverse events in terms of frequency, duration, subjective impact/severity and age at exposure. When necessary, we adopted existing environmental risk measures to include temporal and severity aspects. The environmental information will be related to epigenetic data in order to identify gene-environment signatures.

### Horizontal integration within functional domains across tasks: an outlook

An important asset of our multilevel investigation is the opportunity to examine RDoC-like domains not only within one type of measures but across several types of assessments allowing for a horizontal integration of these constructs across tasks and measurement levels. Three examples of such possible horizontal integration are given below.

#### Acute threat

The fourth version of the RDoC matrix, published in May 2018, (https://www.nimh.nih.gov/research-priorities/rdoc/constructs/rdoc-snapshot-version-4-saved-5-30-18.shtml) categorizes fear learning paradigms and social stress tests as part of the acute threat construct of the negative valence domain. In our study, fear learning and recall tasks are administered in the psychophysiology laboratory whereas the adaptation of the Montreal IST takes place within the MR-scanner (with continuous blood and/or saliva sampling). This allows within-subject comparisons of psychophysiological anticipatory responses to acute threat within a fear learning framework (electric shocks, air puffs) to the more social evaluative acute threat during the stress test on multiple levels. Some measurements such as pulse plethysmography and skin conductance response recordings are obtained during all of these tasks - providing a direct connection, whereas the tasks combined provide a coherent multi-level assessment of the acute stress construct from circuits to physiology, behavior and self-report. In all tasks, we can also map the correlation with polygenic or epigenetic (DNA methylation or microRNA) measures associated with this construct in prior human or animal experiments, allowing integration of molecular information.

#### Arousal

The construct of the arousal and regulatory systems domain will also be captured throughout various tasks and levels, resulting in multiple parameter estimates that can be hierarchically and individually related. Arousal-related processes, for instance hyperarousal, can be quantified by a reduced habituation in the standard psychophysiological measures (e.g., startle responses, SCR) in the psychophysiology lab [126], but also by pupil fluctuations during resting-state fMRI, which are robustly related to activity in the so-called salience network (dACC and bilateral insula, among others) [152]. Such parameter estimates across domains can be related to other relevant levels, including sleep fragmentation as assessed by actigraphy or hyperarousal as obtained through the structured CIDI-interview.

#### Working memory

The cognitive domain provides another instance of horizontal integration, as working memory is assessed both in the MR-scanner and in the neuropsychology laboratory, resulting in behavioral and imaging readouts. Moreover, in the scanner we employ simultaneous pupillometry to assess cognitive load and relate this to a cognitive load specific activity patterns. These physiological, imaging and behavioral data can be related to self-report measures regarding behavioral inhibition and approach as well as to other cognitive control tasks (e.g., response inhibition). These can also be mapped to relevant polygenic risk scores, such as for hippocampal volume for example.

## Summary and outlook

The information collected in BeCOME spans many levels from omics, cellular and imaging data to psychophysiological parameters as well as self-reported symptoms of mental disorders, personality traits and lifetime exposure to trauma and other environmental risk factors. For a more dynamic in-depth phenotyping, BeCOME additionally applies several validated paradigms to experimentally induce a response in a basic system of human functioning (e.g., fear system, stress or reward system) that is frequently disturbed in patients with affective, anxiety and other stress-related mental disorders. The response to these stimulations is read out across multiple levels (mostly with psychophysiological, imaging and stress measurements). The dynamic read-outs can again be related back to basic omics data, environmental risk experiences and other behavioral data.

Extracting information from these multilevel measures using big data approaches, including machine learning methods, may lead to the identification of biology informed diagnoses that could convey information on the specific therapeutic needs of an individual according to their specific dysfunctional pattern. The clinical implication of such results can be illustrated by the previously described example of two patients with opposite symptom patterns but with the same major depression diagnosis. In the future, these two major depression cases might fall within two different disorder classes, one that is mainly characterized by dysregulations in the reward system and another one that is driven by dysfunctions in stress response and the fear system. Such differentiated patient profiles transport more information for an optimized treatment selection than the rather imprecise DSM-based diagnosis of major depression. Besides optimizing the allocation of existing treatments to an individual patient, biology-based subtypes may also lead to the discovery of novel treatment targets and stimulate the development of new pharmaceutical treatments. The overall aim of BeCOME is to contribute to a biology-informed taxonomy of mental disorders that points out the underlying disease mechanism and with it the treatment target. For example, first analyses of the BeCOME reward task and pupillometry data suggest that physiological disturbances in arousal upregulation, when anticipating a reward, might constitute an underlying pathophysiological process of depression symptomatology. Hence, the upregulation and maintenance of arousal during reward anticipation might be a translational process that could prove relevant for stratification of patients, treatment development, and tracking of drug target engagement (Schneider M, Elbau IG, Nantawisarakul T, Pöhlchen D, Brückl T, Erhardt A, et al: The eyes are a window to depression: reduced pupil dilation during reward anticipation in depression, under review). A further gain in knowledge of the biological profile of mental disorders might pave the way for an objective assessment of mental disorders with a validated array of omics and behavioral measures.

### Study status

Recruitment for the BeCOME study was ongoing at the time this manuscript was submitted.

## Data Availability

The datasets generated and/or analysed during the current study contain clinical data and are not publicly available due to the protection of participants’ rights to privacy and data protection but are available from the corresponding author on reasonable request.

## Abbreviations

AUDIT-C: Alcohol use disorder identification test – consumption questions
BeCOME: Biological classification of mental disorders
DSM: Diagnostic and statistical manual of mental disorders
EMG: Electromyographic
fMRI: Functional magnet resonance imaging
ICD: International classification of diseases
IST: Imaging stress test
MPIP: Max Planck Institute of Psychiatry
MRI: Magnet resonance imaging
RDC: Research diagnostic criteria
RDoC: Research domain criteria
SCR: Skin conductance responses
TET: Time estimation task
